# Analysing bioelectrical phenomena in the *Drosophila* ovary with genetic tools: tissue-specific expression of sensors for membrane potential and intracellular pH, and RNAi-knockdown of mechanisms involved in ion exchange

**DOI:** 10.1186/s12861-020-00220-6

**Published:** 2020-07-08

**Authors:** Susanne Katharina Schotthöfer, Johannes Bohrmann

**Affiliations:** RWTH Aachen University, Institut für Biologie II, Abt. Zoologie und Humanbiologie, Worringerweg 3, 52056 Aachen, Germany

**Keywords:** *Drosophila melanogaster*, Oogenesis, Follicle cell, Planar cell polarity, Bioelectricity, Intracellular pH, Membrane potential, GEVI, Ion pump, Ion channel, Gap junction, Innexin, Cytoskeleton, RNAi

## Abstract

**Background:**

Changes in transcellular bioelectrical patterns are known to play important roles during developmental and regenerative processes. The *Drosophila* follicular epithelium has proven to be an appropriate model system for studying the mechanisms by which bioelectrical signals emerge and act. Fluorescent indicator dyes in combination with various inhibitors of ion-transport mechanisms have been used to investigate the generation of membrane potentials (V_mem_) and intracellular pH (pH_i_). Both parameters as well as their anteroposterior and dorsoventral gradients were affected by the inhibitors which, in addition, led to alterations of microfilament and microtubule patterns equivalent to those observed during follicle-cell differentiation.

**Results:**

We expressed two genetically-encoded fluorescent sensors for V_mem_ and pH_i_, ArcLight and pHluorin-Moesin, in the follicular epithelium of *Drosophila.* By means of the respective inhibitors, we obtained comparable effects on V_mem_ and/or pH_i_ as previously described for V_mem_- and pH_i_-sensitive fluorescent dyes. In a RNAi-knockdown screen, five genes of ion-transport mechanisms and gap-junction subunits were identified exerting influence on ovary development and/or oogenesis. Loss of ovaries or small ovaries were the results of soma knockdowns of the innexins *inx1* and *inx3,* and of the DEG/ENaC family member *ripped pocket (rpk)*. Germline knockdown of *rpk* also resulted in smaller ovaries. Soma knockdown of the V-ATPase-subunit *vha55* caused size-reduced ovaries with degenerating follicles from stage 10A onward. In addition, soma knockdown of the *open rectifier K*^*+*^*channel 1 (ork1)* resulted in a characteristic round-egg phenotype with altered microfilament and microtubule organisation in the follicular epithelium.

**Conclusions:**

The genetic tool box of *Drosophila* provides means for a refined and extended analysis of bioelectrical phenomena. Tissue-specifically expressed V_mem_- and pH_i_-sensors exhibit some practical advantages compared to fluorescent indicator dyes. Their use confirms that the ion-transport mechanisms targeted by inhibitors play important roles in the generation of bioelectrical signals. Moreover, modulation of bioelectrical signals via RNAi-knockdown of genes coding for ion-transport mechanisms and gap-junction subunits exerts influence on crucial processes during ovary development and results in cytoskeletal changes and altered follicle shape. Thus, further evidence amounts for bioelectrical regulation of developmental processes via the control of both signalling pathways and cytoskeletal organisation.

## Background

In recent years, bioelectrical signals have been shown to play decisive roles in regulating diverse cellular events [1–6]. For example, in *Drosophila*, a screen of 180 genes identified a variety of ion channels essential for normal wing development [7]. Moreover, in humans, morphological defects caused by mutations in ion-transport mechanisms are associated with so-called channelopathies [8–12]. Accordingly, pre-patterns of membrane potential (V_mem_) and intracellular pH (pH_i_) are supposed to represent a basis for tissue and organ patterning via the control of planar cell polarity and cytoskeletal organisation [3, 5, 13–17].

In *Drosophila*, at least two distinct pathways are responsible for planar cell polarity. One pathway depends on Dsh/Fz and acts in the wing and eye [13], while the second pathway depends on interaction of the cytoskeleton with the extracellular matrix in ovarian follicle cells (FC) [18]. FC display microfilaments at their basal side (bMF) that are oriented perpendicular to the anteroposterior (a-p) axis of the developing follicle [5, 17–19]. Proper bMF-orientation requires integrins as well as planar-polarised distribution of the receptor-tyrosine phosphatase Lar. Lar is known to be involved in signalling between the extracellular matrix and the actin cytoskeleton [20, 21]. The first mutant shown to disrupt polarisation of bMF in FC was called *kugelei,* due to its prominent round-egg phenotype [22].

While bioelectrical phenomena, like gradients of V_mem_ and pH_i_, become increasingly accepted as regulators of development, the mechanisms by which these signals exert influence on developmental pathways are poorly understood. Therefore, it is necessary to identify the ion-transport mechanisms involved in generation and modification of the bioelectrical signals. During *Drosophila* oogenesis*,* the exchange of protons, potassium ions and sodium ions is primarily responsible for stage-specific V_mem_- and pH_i_-patterns as well as for extracellular currents [23–28]. Moreover, in the planar cell-polarity pathway of the *Drosophila* wing and eye, a need for bioelectrical cues to conduct signalling has been demonstrated [13, 29].

The DEG/ENaC-family represents one of the largest ion-channel families in *Drosophila* [30]. In vertebrates, amiloride-sensitive Na^+^-channels have been implicated in some early developmental events, like blocking secondary sperm entry in *Xenopus* eggs or generating the blastocoel [31]. Members of the DEG/ENaC-family mediate Na^+^-absorption across the apical membrane of epithelia; they are essential for Na^+^-homeostasis, and are expressed in gonads and neurons [32–34].

In insects, proton-pumping V-ATPases are located in apical membranes of almost all epithelial tissues, where they energise secondary active transport processes [35, 36]. Moreover, they are responsible for the acidification of cytoplasmic vesicles, e. g., in the follicular epithelium (FE) of *Drosophila* [3, 16, 27]. In *Drosophila* ovarian follicles, an involvement of V-ATPases in bioelectrical phenomena has been supposed [27, 37]. In particular, the asymmetrical accumulation of V-ATPases on one side of the follicle points to a role in regulating spatial coordinates [3, 37]. Several studies demonstrated that V-ATPases are also required for Notch and wingless signalling in *Drosophila* [29, 38, 39].

In *Drosophila* follicles, germline and soma cells are interconnected via gap junctions [40]. Members of the innexin family are known to represent the main gap-junction proteins in invertebrates [41, 42]. In the *Drosophila* ovary, innexins 1 to 4 have been shown to be involved in the formation of different types of gap junctions [43, 44]. Gap junctions can propagate alterations of V_mem_ and pH_i_ between germline and soma cells [3, 40, 44].

In the present study, we used, for the first time, genetically-encoded sensors for V_mem_ and pH_i_ in combination with specific inhibitors of ion-transport mechanisms in order to refine and extend earlier studies using electrophysiological recordings [23, 24] or V_mem_- and pH_i_-sensitive fluorescent dyes [5, 16] in the ovary of *Drosophila*. Out of a large number of available genetically-encoded V_mem_-indicators (GEVIs) with the voltage-sensing domain (VSD) of *Ciona intestinalis*, we chose a member of the ArcLight family. GEVIs of this family display a relatively high sensitivity as well as slow kinetics of activation and inactivation [45, 46]. These characteristics appeared to be useful for analysing slow V_mem_-changes as in the FE of *Drosophila*. The selected pH_i_-sensor comprises a fusion of pHluorin and the Moesin actin-binding domain; it was initially designed for the visualisation of apoptotic cell-phagocytosis [47]. Due to tissue-specific expression, genetically-encoded sensors provide some advantages compared to other methods used to identify ion-transport mechanisms involved in V_mem_- and pH_i_-regulation [48, 49]. In order to refine and extend the knowledge obtained using inhibitors of ion exchange, we performed a RNAi-knockdown screen of genes coding for ion-transport mechanisms and gap-junction subunits that, via V_mem_- and pH_i_-changes in the FE, might have impact on the development of the ovary and/or on oogenesis in *Drosophila*.

## Results

### Genetically-encoded sensors of V_mem_ and pH_i_ reliably respond to inhibitors of ion-transport mechanisms

#### V_mem_-sensor ArcLight and pH_i_-sensor pHluorin-Moesin

Two genetically-encoded fluorescent V_mem_- and pH_i_-sensors, ArcLight and pHluorin-Moesin, in combination with six inhibitors (cf. [5, 16]) were used to analyse the roles that specific ion-transport mechanisms play in regulating V_mem_ and pH_i_ in the follicular epithelium of *Drosophila* during stage S10B.

**Fig. 1:**
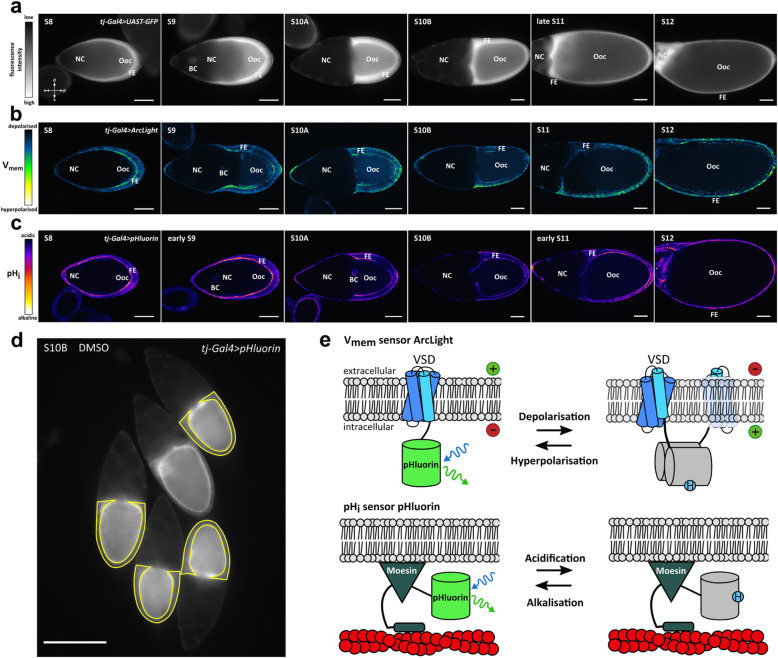
Analysis of V_mem_ and pH_i_ using the genetically-encoded fluorescent V_mem_- and pH_i_-sensors ArcLight and pHluorin-Moesin, respectively. **a** Uniform expression of GFP in the FE using the soma-driver *tj*-Gal4 (control); WFM-images of typical *tj-Gal4 > UAST-GFP* follicles of vitellogenic stages S8-S12 (scale bars represent 50 μm). **b**, **c** Pseudocolour images of follicles expressing ArcLight (**b**) or pHluorin-Moesin (**c**) in the FE; median optical sections (SIM) of typical follicles of S8-S12 (scale bars represent 50 μm). **d** Analysis of fluorescence intensities in the FE (“mean grey value”; area marked in yellow); examples of four follicles of S10B (and one of S11) expressing pHluorin-Moesin (WFM-image; scale bar represents 200 μm). **e** Both the V_mem_-sensor ArcLight and the pH_i_-sensor pHluorin-Moesin use the chromophore pHluorin which responds, in deprotonated state, to blue-light excitation (blue arrow) with the emission of green light (green arrow). The exact mechanism of ArcLight is not known, but is believed to involve voltage-dependent dimerisation leading to protonation of the chromophore (VSD, voltage-sensing domain; scheme inspired by [45]). In cells expressing the respective sensor, relative depolarisation or relative acidification is indicated by weaker fluorescence intensities, whereas relative hyperpolarisation or relative alkalisation is indicated by stronger fluorescence intensities

ArcLight-family GEVIs respond to depolarisation upon blue-light excitation with reduced green fluorescence of superecliptic pHluorin, while they respond to hyperpolarisation with enhanced green fluorescence (Fig. 1e). Superecliptic pHluorin is protonated at relatively depolarised V_mem_ (dark or “ecliptic”) and mostly deprotonated at relatively hyperpolarised V_mem_ (bright). The pH_i_-sensor pHluorin-Moesin emits green light upon blue-light excitation as well. Due to protonation, it responds to relative acidification with reduced fluorescence, whereas, due to deprotonation, relative alkalisation is indicated by enhanced fluorescence (Fig. 1e).

The specific expression of both ArcLight and pHluorin-Moesin at the FC cortex revealed, during the course of vitellogenesis (S8-S12), stage-specific patterns of V_mem_ and pH_i_ (Fig. 1b and c) which are comparable to those obtained previously with the fluorescent indicator dyes DiBAC_4_(3) and 5-CFDA,AM (cf. [3, 5, 16, 17]). Uniform FE-specific expression of the sensors was controlled by the *tj*-Gal4-driven expression of GFP (Fig. 1a).

#### Inhibition of ion-transport mechanisms

**Fig. 2:**
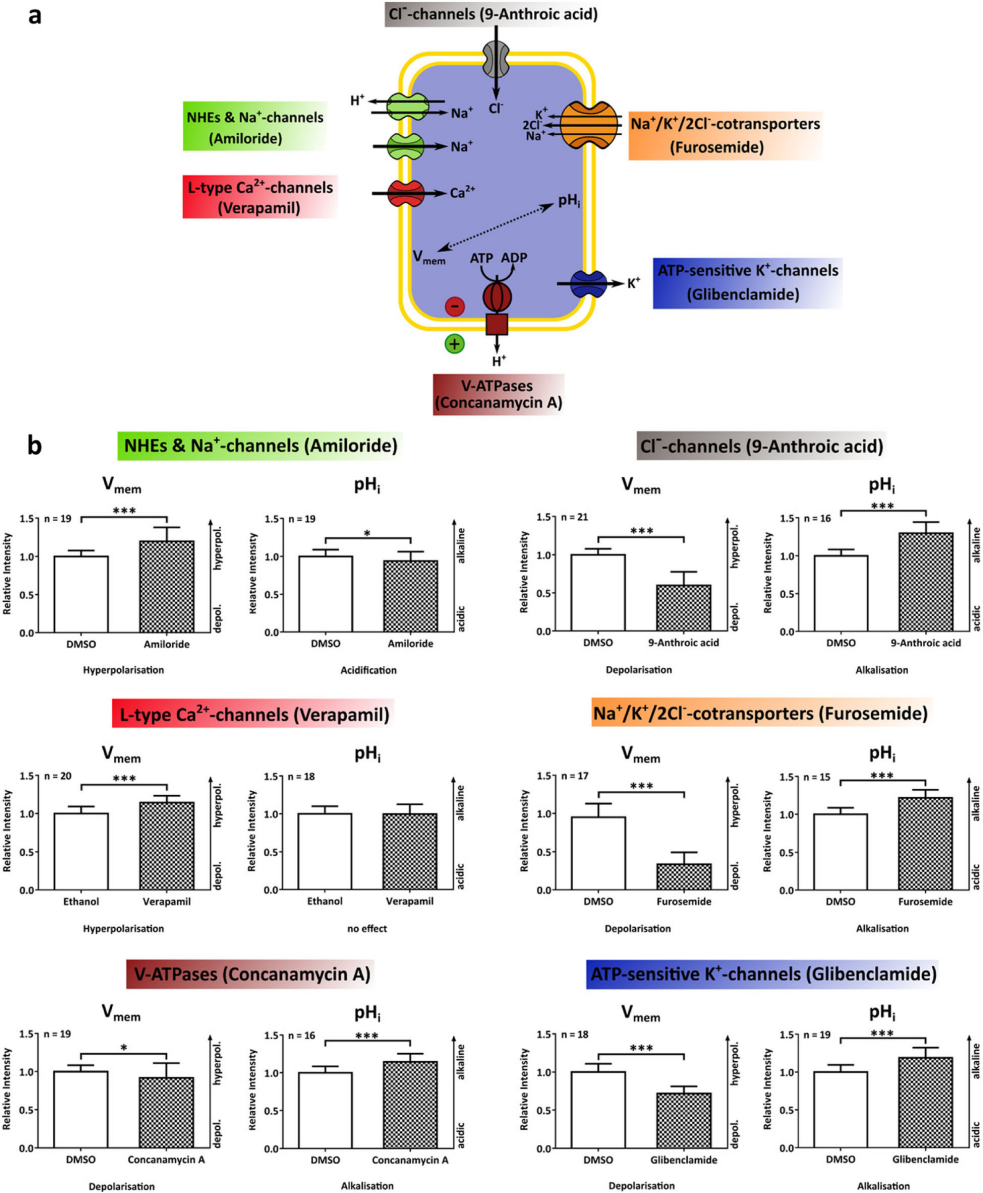
Genetically-encoded V_mem_- and pH_i_-sensors reveal changes of bioelectrical properties resulting from the inhibition of ion-transport mechasms. **a** Schematic overview of the analysed ion-transport mechanisms and their specific inhibitors (in brackets). **b** All inhibitors led to significant changes of V_mem_ and/or pH_i_ in the FE. While inhibition of NHEs and Na^+^-channels or L-type Ca^2+^-channels resulted in relative hyperpolarisation, inhibition of V-ATPases, Cl^−^-channels, Na^+^/K^+−^/2Cl^−^-cotransporters or ATP-sensitive K^+^-channels resulted in relative depolarisation. Concerning pH_i_, inhibition of V-ATPases, Cl^−^-channels, Na^+^/K^+^/2Cl^−^-cotransporters or ATP-sensitive K^+^-channels resulted in relative alkalisation, whereas inhibition of NHEs and Na^+^-channels caused relative acidification. The inhibition of L-type Ca^2+^-channels had no significant effect on pH_i_. Normalised values of 15 < *n* < 21 S10B-follicles were averaged (*relative intensity*). Mean values, shown with their standard deviation, were compared using an unpaired t-test (* *p* < 0.05; *** *p* < 0.001)

**Fig. 3:**
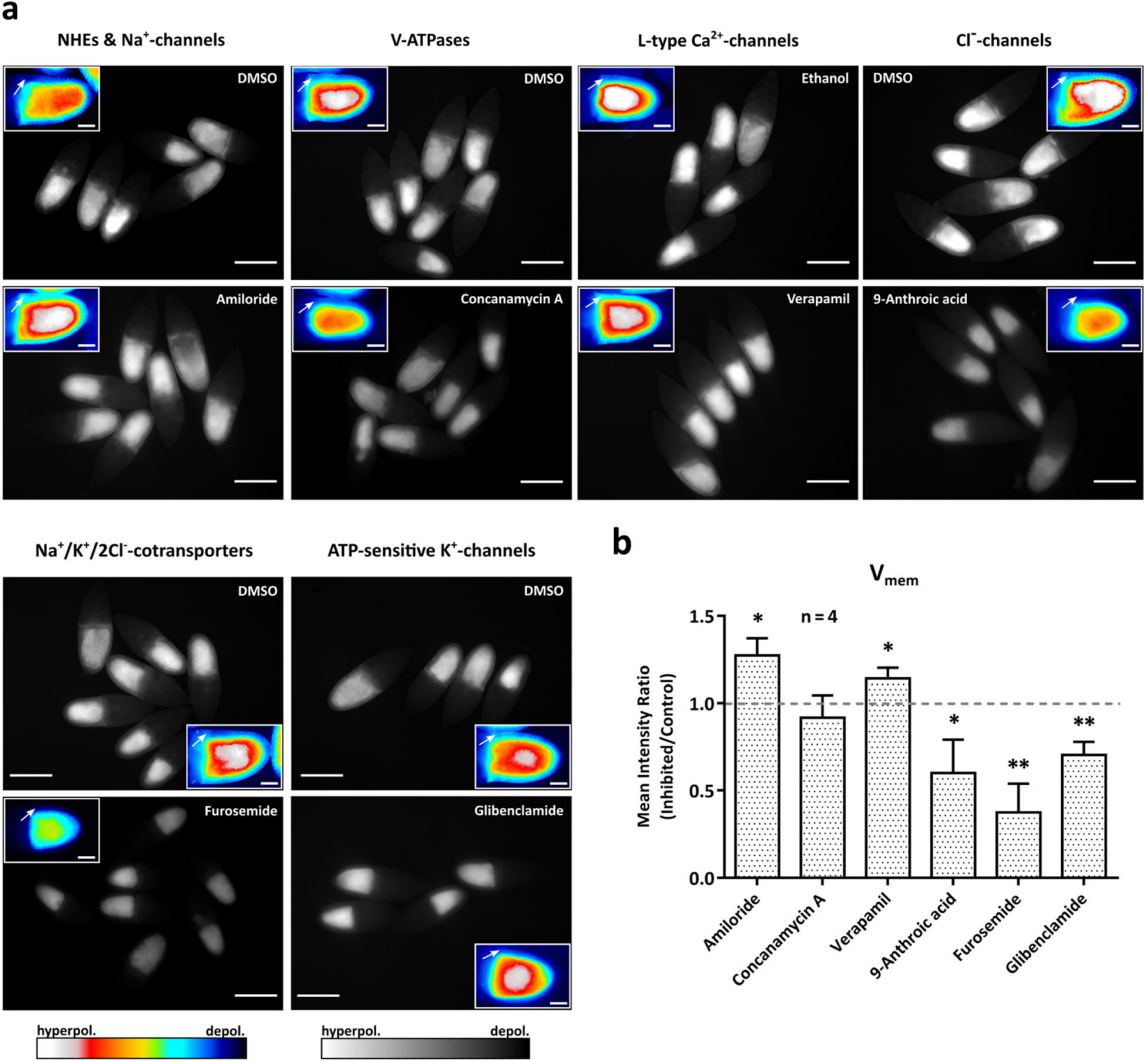
Influences of inhibitors of ion-transport mechanisms on V_mem_ in the FE. **a** WFM-images of typical experiments showing ArcLight-expressing S10B-follicles after incubation in either the respective inhibitor or the control solution (DMSO or ethanol; scale bars represent 200 μm); insets show enlarged examples of representative follicles in pseudocolour (arrows point to FE; scale bars represent 50 μm). Relative hyperpolarisation is indicated by stronger (bright/white), relative depolarisation by weaker (dark/blue) fluorescence intensities. The experiments were repeated at least four times. **b** While amiloride and verapamil caused increasing fluorescence intensities (hyperpolarisation), 9-anthroic acid, furosemide, glibenclamide and concanamycin A caused decreasing fluorescence intensities (depolarisation). To consider the variability between experiments, *mean intensity ratios* of the experimental and control groups (*inhibited/control*) of *n* = 4 experiments for each inhibitor were calculated. Mean values, shown with their standard deviation, were compared using a one-sample t-test (* *p* < 0.05; ** *p* < 0.01). The strongest effects on V_mem_ were obtained with 9-anthroic acid, furosemide and glibenclamide, respectively

**Fig. 4:**
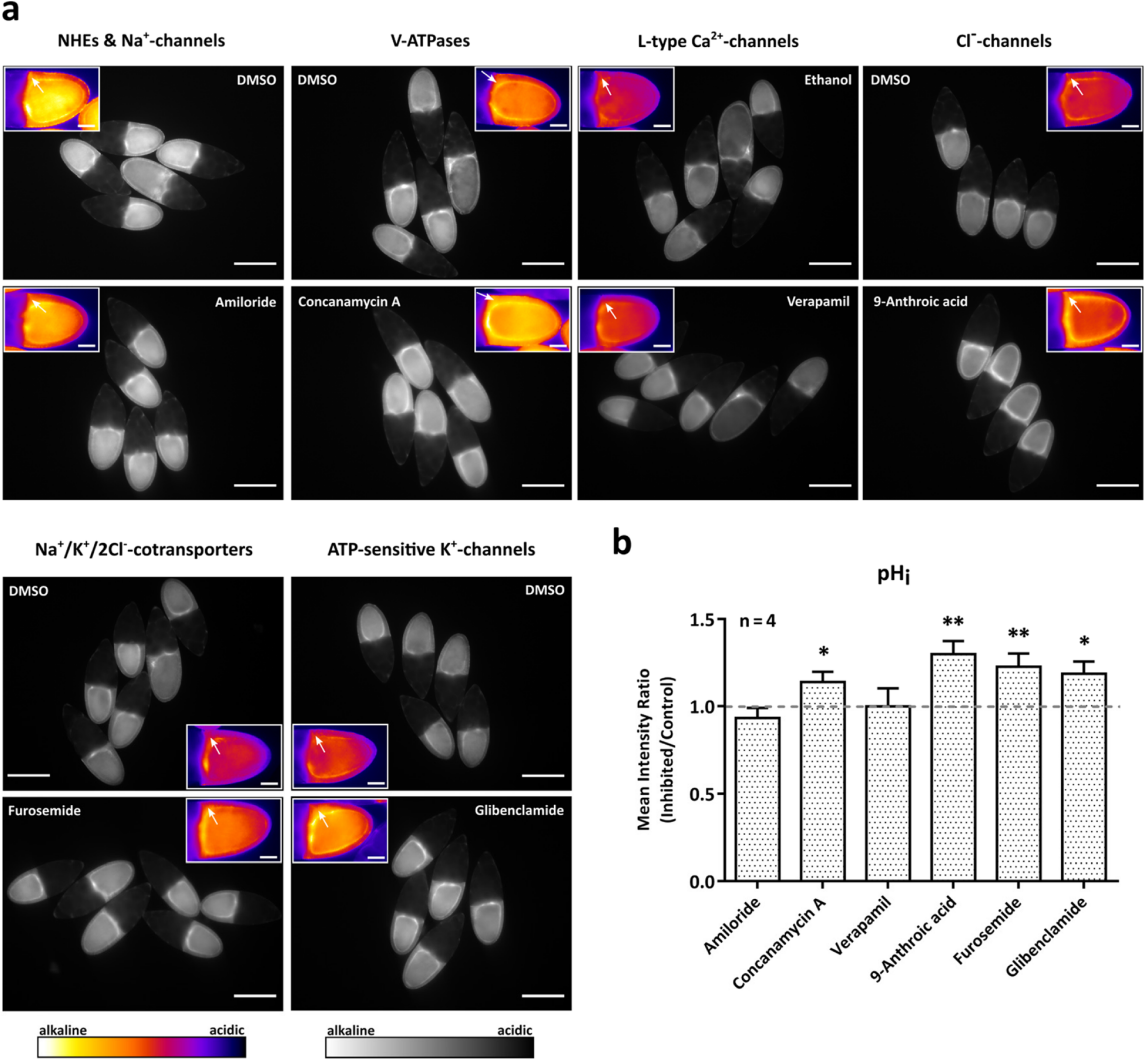
Influences of inhibitors of ion-transport mechanisms on pH_i_ in the FE. **a** WFM-images of typical experiments showing pHluorin-Moesin-expressing S10B-follicles after incubation in either the respective inhibitor or the control solution (DMSO or ethanol; scale bars represent 200 μm); insets show enlarged examples of representative follicles in pseudocolour (arrows point to FE; scale bars represent 50 μm). Relative alkalisation is indicated by stronger (bright/white), relative acidification by weaker (dark/blue) fluorescence intensities. The experiments were repeated at least four times. **b** While amiloride caused a slight decrease in fluorescence intensity (acidification), concanamycin A, 9-anthroic acid, furosemide and glibenclamide led to significantly increasing fluorescence intensities (alkalisation). Verapamil showed no effect on pH_i_. To consider the variability between experiments, *mean intensity ratios* of the experimental and control groups (*inhibited/control*) of *n* = 4 experiments for each inhibitor were calculated. Mean values, shown with their standard deviation, were compared using a one-sample t-test (* *p* < 0.05; ** *p* < 0.01). The strongest effects on pH_i_ were obtained with concanamycin A, 9-anthroic acid, furosemide and glibenclamide, respectively

Resulting from the specific inhibition of ion-transport mechanisms, both genetically-encoded sensors report changes of bioelectrical properties in the FE (Figs. 1d, 2a and b, 3b and 4b). While, in the ArcLight-expressing FE, the inhibitors amiloride (NHEs, Na^+^-channels) and verapamil (voltage-dependent L-type Ca^2+^-channels) led to higher fluorescence intensities (hyperpolarisation), the inhibitors concanamycin A (V-ATPases), 9-anthroic acid (Cl^−^-channels), furosemide (Na^+^/K^+^/2Cl^−^-cotransporters) and glibenclamide (ATP-sensitive K^+^-channels), respectively, led to lower fluorescence intensities (depolarisation). While the strongest effect on V_mem_ was observed with furosemide, the weakest was observed with concanamycin A (Fig. 2b).

In the pHluorin-expressing FE, the inhibitors concanamycin A, 9-anthroic acid, furosemide and glibenclamide, respectively, led to higher fluorescence intensities (alkalisation), whereas amiloride led to lower fluorescence intensity (acidification). While the strongest effect on pH_i_ was observed with 9-anthroic acid, verapamil showed no significant effect (Fig. 2b).

To directly compare the effects of all inhibitors on either V_mem_ or pH_i_, a *mean intensity ratio* of the experimental and the control groups of four experiments was calculated for each treatment (Figs. 3 and 4). This evaluation considered the variability between experiments with the same treatment, whereas the evaluation shown in Fig. 2 considered the variability between different follicles. Both evaluations disclosed inhibitory effects with the same tendency on V_mem_ and pH_i_. In addition, they confirmed the results of previous studies [3, 5, 16] showing that the targeted ion-transport mechanisms are involved in the regulation of bioelectrical properties in the FE of *Drosophila.*

### RNAi-knockdowns of ion-transport mechanisms and gap-junction subunits affect ovary development and oogenesis

**Fig. 5:**
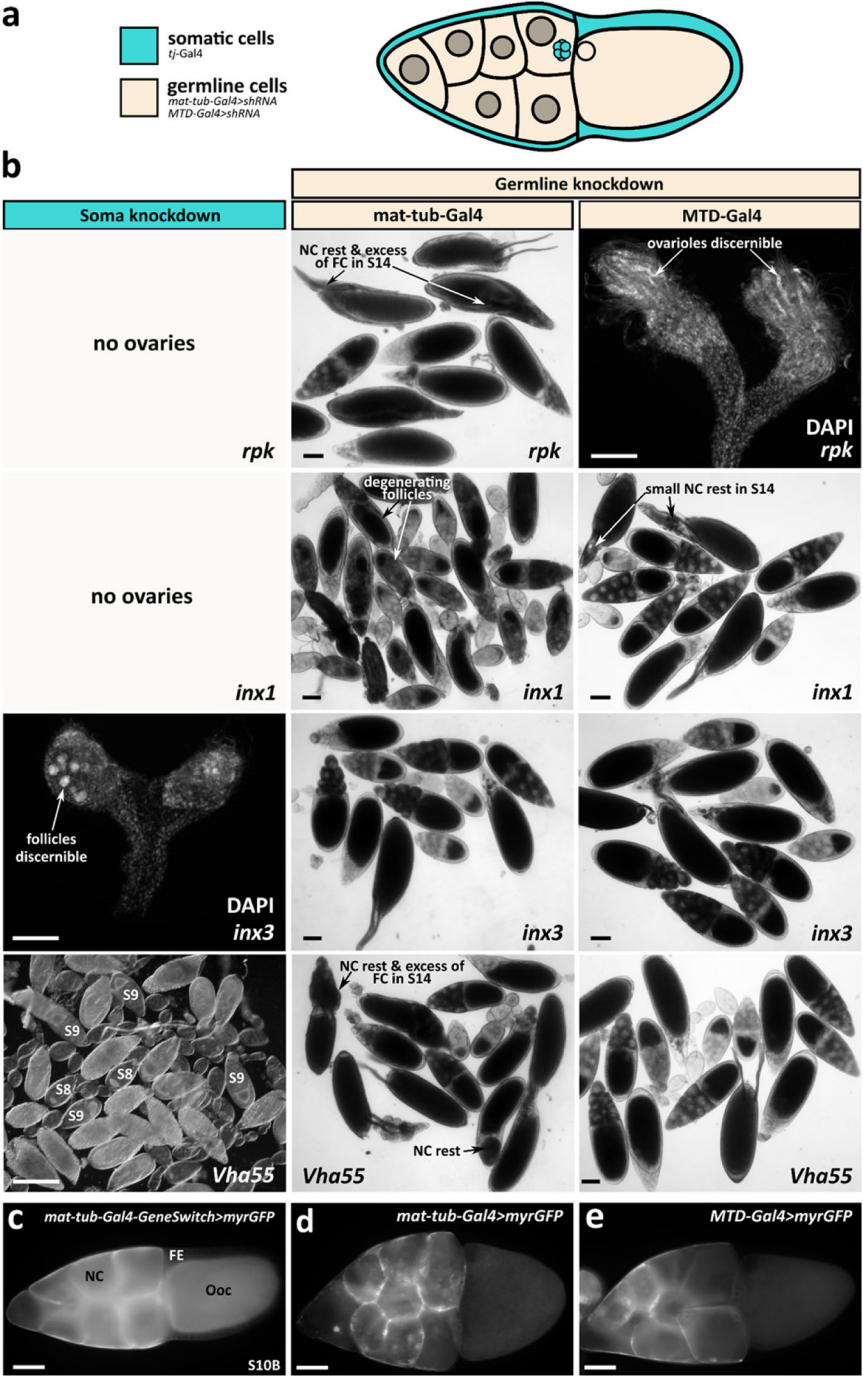
Summary of genes coding for ion-transport mechanisms and gap-junction subunits showing effects in RNAi-knockdown. **a** Scheme of a S10B-follicle: somatic cells (turquoise), germline cells (beige). For soma knockdown of relevant genes of ion-transport mechanisms and gap-junction subunits, the *tj*-Gal4 driver was used, whereas for germline knockdown, the mat-tub-Gal4 and MTD-Gal4 drivers were used. **b** Out of all performed RNAi-knockdowns (left column: soma knockdown via *tj*-Gal4; middle and right columns: germline knockdown via mat-tub-Gal4 and MTD-Gal4, respectively), the knockdowns of *rpk* (first line), *inx1* (second line), *inx3* (third line; left: size-reduced ovaries with single follicles, middle and right: no effects), and *vha55* (fourth line; right: no effects) resulted in striking effects on ovary morphology (DAPI) and/or oogenesis (scale bars represent 100 μm). While soma knockdown of *vha55* led to degenerating follicles from S10A onward, germline knockdown of *inx1*, *vha55* and *rpk* resulted in NC rests and excess of FC around micropyle. The strongest effects were observed for *rpk*: Reduced *rpk*-transcript levels in the FE resulted in loss of ovaries, whereas reduced levels in the germline led to size-reduced paired or single ovaries showing ovarioles, but no follicles. **c**-**e** Germline driver-directed expression of myrGFP in plasma membranes of NC and Ooc (**c**: mat-tub-Gal4-GeneSwitch, **d** mat-tub-Gal4, **e** MTD-Gal4). myrGFP has an N-terminal myristoylation sequence directing GFP to plasma membranes under UASp-control. For soma driver-directed expression of GFP, see Fig. 1a

**Fig. 6:**
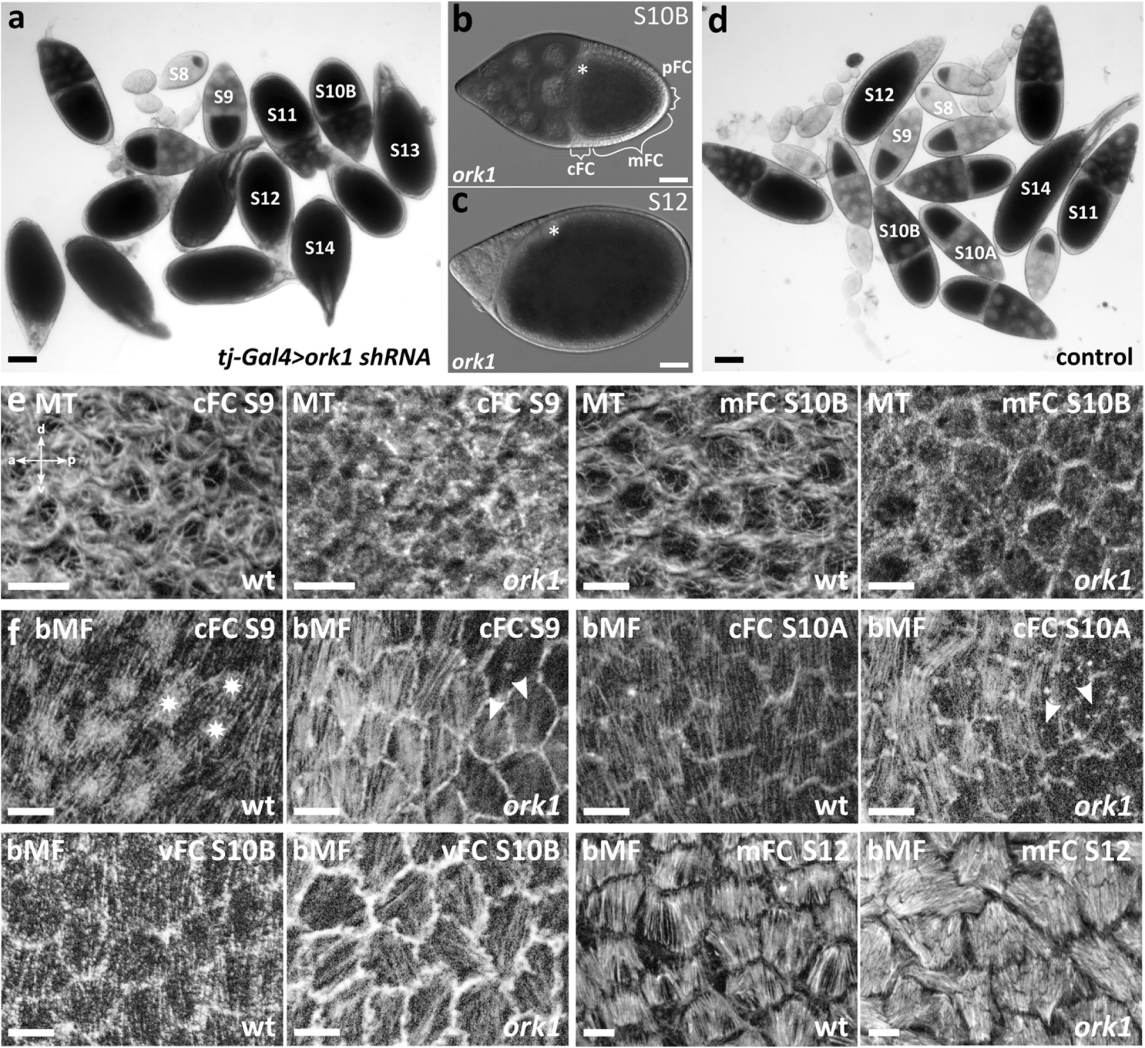
RNAi-knockdown of *ork1* in the FE results in spherical follicles with altered cytoskeletal organisation. **a**-**d** Brightfield-images of follicles from *tj-Gal4 > ork1 shRNA* (**a**-**c**) and *mat-tub-Gal4-GeneSwitch > ork1 shRNA* ovaries (control; **d**). **a** For soma knockdown of *ork1*, the *tj*-Gal4 driver was used. Ovaries of all analysed flies contained spherical follicles and eggs (S8-S14; scale bar represents 100 μm). **b** and **c** Brightfield-images of S10B- and S12-*tj-Gal4 > UAS-ork1 shRNA* follicles; the oocyte nucleus (dorsal) is marked with an asterisk (scale bars represent 50 μm). **d** Ovaries from *mat-tub-Gal4-GeneSwitch > UAS-shRNA* flies, having a low transcription level of *ork1*-shRNA, were used as control (scale bar represents 100 μm). Similar to ovaries from strong germline knockdowns via the mat-tub-Gal4 and MTD-Gal4 drivers (cf. Table 1), ovaries from control flies only produced follicles resembling the wt. **e** In contrast to wt, *ork1*-follicles (S9 and S10B) exhibit a weaker microtubule (MT) cytoskeleton in the FE, and the MT are not aligned along the a-p axis (scale bars represent 10 μm). Tangential optical sections (SIM) of typical anti-acetylated α-tubulin-treated follicles are shown. **f** Concerning basal microfilaments (bMF) in the FE, *ork1* exhibits even stronger anomalies in comparison to wt. Tangential optical sections of typical S9, S10A, S10B and S12 wt- and *ork1*-follicles stained with fluorescent phalloidin are shown (cFC, centripetal FC; mFC, mainbody FC; vFC, ventral FC; a, anterior; p, posterior; d, dorsal; v, ventral); d-v orientation, as indicated, applies to all images, except vFC-images (scale bars represent 10 μm). Since, due to bMF-condensations in wt-follicles, it is difficult to reveal transversal bMF-alignment in dorsal and lateral cFC [5, 17], vFC are shown for S10B. Typical bMF-condensations (asterisks), as in S9 cFC in wt, are missing in *ork1*. The bMF in *ork1* S10A show the same parallel alignment as in wt, however, in some areas (arrowheads), the bMF-cytoskeleton is weaker. During S10B and S12, *ork1-*follicles are characterised by a disturbed transversal transcellular bMF-alignment, resembling the *kugelei* mutant [22]

The purpose of our screen was to investigate whether RNAi-knockdowns of candidate genes of ion-transport mechanisms or gap-junction subunits result in long-term effects on ovary development and/or on oogenesis. In particular, we wanted to see if RNAi-knockdowns exert influence on the FE-specific cytoskeleton in a similar way as various inhibitors of ion-transport mechanisms [16]. For RNAi-knockdown in the FE, we combined VDRC UAS-strains or TRIP UAS-lhRNA- and UAS-shRNA-strains of relevant genes with the soma-specific *tj*-Gal4 driver line. In addition, we used the germline-specific mat-tub-Gal4 or MTD-Gal4 driver lines for RNAi-knockdown in NC and Ooc (see Fig. 5, Table 1 and Additional file: Table S1). As controls, ovaries from flies expressing the UAS-constructs at low levels in the germline were used (e. g., *mat-tub-Gal4-GeneSwitch > ork1 shRNA*). As expected, these ovaries did not show any phenotype differing from wt (Fig. 6d).

**Table 1:** Genes of ion-transport mechanisms and gap-junction subunits showing effects following RNAi-knockdown

Stock ID	Gene name	Protein function	Phenotype Soma	Germline
**Proton pumps**				
VDRC46553	*vha55*	V-type H^+^-ATPase subunit	size-reduced ovaries, degeneration > S10A	not determined
BL40884	*vha55*		no effects	S11-S14 with NC rests, S14 with excess of FC around micropyle^b^
**Sodium channels**				
BL39053	*rpk (dGNaC1)*	DEG/epithelial sodium channel	no ovaries	small ovaries, ovarioles w/o follicles^a^, S11-S14 with NC rests, S14 with excess of FC around micropyle
**Potassium channels**				
BL53994	*ork1*	Open-rectifier potassium channel	spherical follicles (resembling *kugelei* mutant), alterations of basal cytoskeleton in FC (bMF & MT)	no effects^ab^
**Innexins**				
BL44048	*inx1*	Gap-junction subunit	no ovaries	S14 with NC-anomalies^a^, many degenerating follicles^b^
BL60112	*inx3*	Gap-junction subunit	small ovaries, no ovarioles, few follicles	no effects^ab^

At least 10 females were scored for each strain

*BL* Bloomington *Drosophila* Stock Center number, *VDRC* Vienna *Drosophila* Resource Center number

Soma driver: *tj*-Gal4, ^a^ germline driver: MTD-Gal4, ^b^ germline driver: mat-tub-Gal4

We identified five genes of ion-transport mechanisms and gap-junction subunits showing effects on ovary development and/or on oogenesis (Table 1, Figs. 5 and 6): RNAi of *vha55* (subunit B of V-ATPase) caused, via soma knockdown, size-reduced ovaries with degenerating follicles from S10A onward or, via germline knockdown (depending on the RNAi-construct), NC rests in S11-S14 and excess of FC around the micropyle in S14. Complete loss of ovaries or size-reduced ovaries (some follicles but no ovarioles discernible) were the results of soma knockdowns of *inx1* or *inx3* (innexin; gap-junction subunit). In addition, germline knockdown of *inx1* (via mat-tub-Gal4) led to degenerating follicles of all vitellogenic stages.

### *RNAi-knockdowns of the genes rpk and ork1 show striking effects*

The strongest RNAi-knockdown effects were observed for *rpk (ripped pocket)*, a member of the DEG/ENaC (epithelial sodium-channel) family: Reduced transcript levels of *rpk* in the FE resulted in complete loss of ovaries, whereas reduced levels in the germline (via MTD-Gal4) led to size-reduced paired or single ovaries showing discernible ovarioles, but no follicles. Via the mat-tub-Gal4 driver, follicles of S11-S14 with NC rests, and follicles of S14 with excess of FC around micropyle were obtained.

Females with RNAi-knockdown of *ork1 (open-rectifier K*^*+*^*channel 1)* in the soma produced spherical follicles, resembling the *kugelei* mutant (Fig. 6a-c, cf. [22]). This phenotype was especially prominent in follicles older than S10B. Compared to wt, *ork1*-follicles revealed alterations in the organisation of the bMF- and MT-patterns in the FE (Fig. 6e-f).

As described previously [5, 17, 19], the bMF of wt-follicles are polarised perpendicular to the a-p axis (transversal alignment), especially during S8, S10A and S12. On the other hand, the MT of wt-follicles are characterised by a-p alignment in centripetal FC (cFC) in S9, as well as in cFC and mainbody FC (mFC) in S10B.

In *ork1* follicles, however, no a-p alignment of MT was detected in any analysed stage, while the overall MT-pattern is less dense and less polarised compared to wt (Fig. 6e). On the other hand, typical condensations of bMF (Fig. 6f), as in wt cFC in S9, are missing in *ork1*. Although bMF-bundles in *ork1* S10A show the same parallel transversal alignment as in wt, the overall bMF-cytoskeleton appears to be weaker in some areas. In contrast to wt, *ork1* S10B and S12 are characterised by disturbed transversal bMF-alignment, showing parallel bundles within FC, but chaotic organisation relative to neighbouring FC. The degrees of cytoskeletal alterations vary between different *ork1-*follicles of the same stage and between different areas in the same follicle. Taken together, during vitellogenic stages, wt-follicles show characteristic longitudinal MT- and transversal bMF-alignments and an elongated follicle shape (cf. [5, 17]). In contrast, *ork1*-follicles are characterised by disturbed MT- and bMF-alignments and a spherical follicle shape, resembling the cytoskeletal organisation and follicle shape in round-egg mutants [18, 22, 50].

## Discussion

### V_mem_- and pH_i_-changes in the FE revealed by tissue-specifically expressed sensors

We have shown that the genetically-encoded sensors ArcLight and pHluorin-Moesin respond to bioelectrical changes occurring in the FE during the course of oogenesis. Moreover, in the FE of S10B, both sensors revealed changes of V_mem_ or pH_i_ resulting from the inhibition of several ion-transport mechanisms that have been characterised in previous studies using various methods [3, 16, 25–27, 34, 51, 52]. Thus, our study shows that genetically-encoded sensors are reliable tools for investigations of this kind. In addition, the results lend further support to the notion that NHEs, Na^+^-channels, V-ATPases, ATP-sensitive K^+^-channels, voltage-dependent L-type Ca^2+^-channels, Cl^−^-channels, and Na^+^/K^+^/2Cl^−^-cotransporters play important roles in modifying V_mem_ and pH_i_ in the FE of *Drosophila*.

While the strongest effect on V_mem_ was observed using furosemide (Na^+^/K^+^/2Cl^−^-cotransporters), the weakest was observed using concanamycin A (V-ATPases). The strongest effect on pH_i_ was obtained with 9-anthroic acid (Cl^−^-channels), whereas verapamil (L-type Ca^2+^-channels) showed no significant effect. Relatively small impact of inhibitors, as observed e. g. for concanamycin A or verapamil, is supposed to be due to compensatory effects exerted by other ion-transport mechanisms. Especially members of the V-ATPase- and DEG/ENaC-families [30, 33, 36] can substitute for other family members as well as for other types of ion-transport mechanisms.

Using the genetically-encoded sensors, we detected similar inhibitory effects on V_mem_ and pH_i_ in the FE as described previously using the voltage- and pH-sensitive fluorescent dyes DiBAC_4_(3) and 5-CFDA,AM, respectively [16, 48, 49]. According to both methods, the treatment with glibenclamide, furosemide or 9-anthroic acid resulted in alkalisation. Glibenclamide (ATP-sensitive K^+^-channels) is supposed to block H^+^-transport indirectly [16, 35], while furosemide and 9-anthroic acid are expected to influence pH_i_ via Cl^−^/HCO_3_^−^-antiport [6, 16, 53, 54]. For concanamycin A, inhibiting V-ATPases [55], we observed alkalising effects in the FE. For bafilomycin A1, another inhibitor of V-ATPases, alkalisation of cytoplasmic vesicles and acidification of the cytoplasm was reported [16]. Therefore, the alkalisation observed for concanamycin A is supposed to refer to cytoplasmic vesicles not discernible with pHluorin-Moesin using WFM.

Instead of hyperpolarisation, as reported by [16], we observed strong depolarisation after treatment with 9-anthroic acid, furosemide, glibenclamide or concanamycin A. In the case of DiBAC_4_(3), reduced fluorescence intensity, indicating hyperpolarisation, might also be due to quenching [49], since depolarising effects of glibenclamide or bafilomycin A1 have been described [56, 57]. On the other hand, in our experiments, higher inhibitor concentrations (up to × 100, compared to [16]) were necessary to reliably detect V_mem_- and pH_i_-changes with the membrane-bound genetically-encoded sensors. The observed depolarisation might, therefore, be attributed to high inhibitor concentrations representing a challenge for the cell. Correspondingly, blockers of oxidative phosphorylation and, thus, of almost all energy-dependent ion transport, like sodium azide or dinitrophenol (cf. [25]), had also depolarising effects on the FE (unpublished results).

Compared to fluorescent indicator dyes, one disadvantage of membrane-bound genetically-encoded sensors is their lower sensitivity, making longer exposure times and higher inhibitor concentrations necessary. Apart from that, these sensors provide several advantages: In combination with the Gal4-UAS-system, they allow the visualisation of V_mem_- or pH_i_-changes in the cell type of choice without any influences from adjacent cell types. In addition, due to stable expression and low sensitivity to photo-bleaching, long-term imaging studies are more practicable. Finally, since unintentional interactions with other substances, as possible for fluorescent dyes, are reduced, shorter experimental protocols can be applied [45, 58, 59].

In conclusion, the use of genetically-encoded sensors and fluorescent indicator dyes [16] both revealed alterations of V_mem_ and/or pH_i_ in the FE. Therefore, both methods provide evidence that the targeted ion-transport mechanisms play important roles in generating bioelectrical signals during oogenesis of *Drosophila*.

### RNAi-knockdowns of a DEG/ENaC-subunit, a V-ATPase-subunit, or gap-junction subunits exert long-term effects on ovary development and/or oogenesis

Due to results from inhibitor studies, it was tempting to investigate whether RNAi-knockdowns of candidate genes of ion-transport mechanisms or gap-junction subunits, showing enriched ovary expression, affect the course of ovary development or oogenesis. We found highly penetrant phenotypes for the genes *rpk*, *vha55*, *inx1* and *inx3*. Most severe effects were obtained after RNAi-knockdown in somatic cells, indicating that the respective proteins are particularly relevant in FC.

Several ion-transport mechanisms have already been related to pH_i_-regulation in the *Drosophila* ovary. It has been reported that the Na^+^/H^+^-exchanger Nhe2 is responsible for an increase in pH_i_ during prefollicular cell differentiation [6]. In addition, ae2, a Cl^−^/HCO_3_^−^-exchanger, was identified as a regulator of pH_i_ in the FC lineage: Loss of *ae2* resulted in reduced fertility, fewer ovarioles, reduced follicle number and reduced ovary size, suggesting that this phenotype is caused by dysregulation of pH_i_ [6]. Data from our RNAi-screen indicate an impairment of ovary development and/or oogenesis after knockdown of the DEG/ENaC-subunit RPK, the V-ATPase-subunit Vha55, and the gap-junction subunits Inx1 and Inx3.

### *DEG/ENaC-subunit RPK*

The strongest effects after both soma- and germline-knockdown were observed for *rpk*. It has been reported that *rpk* is specifically expressed in gonads and in the early embryo, having a proposed function in gametogenesis [31, 34, 60]. Consequently, soma-knockdown of *rpk* resulted in complete loss of ovaries, whereas germline-knockdown led to size-reduced paired or single ovaries with beginning ovariole formation, but no developing follicles. The severe phenotypes following *rpk*-knockdown are likely to be related with functions during larval development.

Many genes of the *pickpocket* family, like *rpk*, exhibit changing expression patterns throughout early development as well as in adult females, providing further hints for their role in developmental signalling and morphogenesis [6, 30]. Microarray-expression data from the FlyAtlas database indicate highest expression levels for *rpk* in ovary and testis [30]. However, *rpk* was not detected in ovarian stem cells and early cysts [34]. It has been suggested that *rpk* and related genes play a role in fluid distribution and cell-volume regulation during gametogenesis and early development [33]. Defects in volume regulation of NC and FC would explain the occurrence of NC rests and the excess of FC in S14.

### *V-ATPase-subunit Vha55*

Soma-knockdown of *vha55*, coding for subunit B of the vacuolar H^+^-ATPase, caused size-reduced ovaries with degenerating follicles from stage S10A onward. It has been reported that genetic knockout of *vha55* leads to a larval lethal phenotype [61]. V-ATPases are highly expressed in ovaries [36, 62] where they are predominantly located in apical FC membranes and in the oolemma [27, 37]. Moreover, V-ATPases are presumed to be involved in bioelectrical phenomena during oogenesis [3, 27] as well as in osmoregulation and follicle growth by water uptake, especially during S10-S12 [27]. Due to the loss of Vha55-function, follicle growth might be inhibited in S10 and, as a consequence, degeneration might take place. On the other hand, organelle-associated V-ATPases are necessary for the acidification of cytoplasmic vesicles (cf. [5, 27]). Consequently, cells lacking V-ATPase-function show impaired acidification of the endosomal compartment and fail to degrade endocytic cargoes [38]. This observation could also explain the degeneration during S10, since cargo sorting is essential for epithelial polarisation, vitellogenesis and other developmental processes [63].

### *Gap-junction subunits Innexin 1 and Innexin 3*

For *inx1,* a function in somatic stem-cell formation is likely since no ovaries were found after soma-knockdown. Moreover, *inx1* has been shown to be predominantly expressed in FC [44]. For mutants of another gap-junction gene, *inx4*, it has been reported that size-reduced gonads correlate with reduced survival of differentiating early germline cells [42, 64]. Our *inx3*-soma knockdown resulted in size-reduced ovaries, in which few follicles were discernible. Therefore, we assume influences of *inx3* on FC differentiation. Rudimentary ovaries combined with impaired follicle maturation, as observed for *inx3*, are also found in several mutants, e. g., the transcription-factor mutant *stonewall* [65]. After germline-knockdown of *inx3*, no defects were observed, which corresponds to the predominant expression of *inx3* in FC [44]. Considering that bioelectrical signals can pass, via gap junctions, from somatic cells to germline cells and vice versa [40], changes of V_mem_ and pH_i_, resulting from RNAi-knockdown either in the soma or the germline, might also become transmitted to the connected tissue and exert indirect influence on development.

Our RNAi-knockdowns of *inx2* and *ductin* had no effects on ovary or follicle morphology (see Additional file: Table S1). Previously, *inx2* has been associated with defects in gametogenesis, and *ductin*, subunit c of V-ATPase, was expected to contribute to developmentally important bioelectrical signals [37, 44, 66–68]. Such missing effects of RNAi-knockdown might depend on the respective RNAi-strain used since, e. g., not all tested *rpk*- or *ork1*-strains caused knockdown-effects (Additional file: Table S1). Similarly, it has been reported that loss of *stim*-transcripts caused severe wing defects and resulted in size-reduced wings [7]. However, in our screen, no effects of *stim*-knockdown could be detected in the ovary (Additional file: Table S1).

### RNAi-knockdown of the potassium channel Ork1 results in spherical follicles with altered cytoskeletal organisation in the FE

As a knockdown-candidate for K^+^-channels, we chose the gene *ork1 (open rectifier K*^*+*^*channel 1)*. According to the FlyAtlas database of gene expression [52], *ork1* RNA is enriched in the ovary. While soma-knockdown of *ork1* resulted in altered follicle shape, germline-knockdown had no effect.

Analysis of the bMF-organisation in the FE revealed cytoskeletal peculiarities in *ork1*-follicles compared to wt. Wt-follicles show transversal bMF-alignment in S8-S12 (cf. [5]) and an elongated shape, whereas *ork1*-follicles show disturbed bMF-alignment and a spherical shape. Similar to the round-egg mutants *fat2/kugelei*, *trc, fry, msn* and *Lar* [18, 20–22, 50, 69], the failure to globally organise bMF in *ork1* correlates with the failure of follicles to elongate along the a-p axis. Accordingly, it has been proposed that the planar-polarised bMF-pattern in wt provides a molecular corset restraining the increase in size along the transversal axis and contributing to follicle elongation [18, 22]. It is known that Lar, a receptor tyrosine phosphatase, interacts with extracellular matrix proteins as well as with the bMF-cytoskeleton and is required for polarised bMF-organisation [18, 20, 21]. Consistent with this, mutants of LanA, a component of the extracellular matrix being polarised perpendicular to the a-p axis of the follicle, produce round eggs as well [21, 22]. Moreover, a screen for round-egg mutants revealed a possible function of the Nuclear Dbf2-related (NDR) kinase Tricornered (Trc) in regulating either bMF, cell-extracellular matrix interactions or transcription-factor activity [50]. Trc and its activator Fry, and Msn, a presumed upstream activating kinase of Trc, are also required for planar cell polarity in the FE at early stages of follicle elongation [50]. In addition, cell-cell communication is needed for the planar polarisation of bMF in FC, since mutations in the atypical cadherin *fat2* (allele of *kugelei*) show a particularly strong round-egg phenotype [18].

Considering that all these genes are part of a pathway establishing planar cell polarity in the FE, we assume a function for *ork1* in the same pathway. Since planar-polarised bMF-orientation requires the orchestrated action of a large number of FC [18], the variability (within a follicle as well as between follicles) of bMF-orientation after soma-knockdown of *ork1* seems reasonable. Consistent with the fact that the round-egg phenotype of *ork1* is especially prominent in later developmental stages, the follicle-shape defects in mutants of *trc*, *fry* or *kugelei* are prominent not before S10 [22, 50]. As consequence of the *ork1*-knockdown, we also observed a disturbed MT-alignment along the a-p axis as well as a weaker MT-cytoskeleton in general. In insects, a polarised MT-pattern in the FE has long been associated with the control of egg shape [70].

As current knowledge about planar FC polarity and follicle elongation comes predominantly from the described round-egg mutants, the involvement of ion-transport mechanisms, like Ork1, adds new insight into these processes. A role for Nhe2 in Fz-mediated planar cell polarity signalling has already been reported [13]. The authors suggested a model in which Dsh binds weakly to Fz, and the proximity of Fz to Nhe2 helps to maintain a slightly basic local pH_i_ which facilitates the interaction of Dsh and Fz. Under acidic conditions, however, this interaction is weakened, leading to a repulsion of Dsh from Fz [13]. Moreover, the V-ATPase-subunit VhaPRR has been identified as a regulator of Wingless and planar cell-polarity signalling: VhaPRR could promote a favourable pH_i_-environment that supports Fz-signalling, alters Fz-conformation, promotes assembly or regulates Fz-trafficking [29].

The V_mem_- and/or pH_i_-dependent binding and surface recruitment of signalling-pathway components is one possible way how bioelectrical signals, generated by ion-transport mechanisms, exert influence on signalling pathways. Therefore, we propose that *ork1*, besides its other reported functions [71, 72], is involved, via bioelectrical signalling, in the establishment of planar cell polarity in the FE, thereby contributing to an elongated follicle shape. This interpretation is in accordance with previous studies suggesting influences of V_mem_- and pH_i_-changes on cytoskeletal organisation and planar cell polarity [5, 16]. Moreover, correlations between alterations in bioelectrical patterns and changes in planar cell polarity were recently described in the mutant *gurken* [17].

## Conclusion

The genetic tool box of *Drosophila* provides several means for a refined and extended analysis of bioelectrical phenomena. Both the V_mem_-sensor ArcLight, initially designed to track action potentials in neurons, and the pH_i_-sensor pHluorin-Moesin, initially designed to analyse phagocytosis, are useful tools to investigate tissue-specific bioelectrical properties during oogenesis. In comparison to fluorescent indicator dyes, genetically-encoded sensors provide several technical and practical advantages. For some types of experiments, however, the use of indicator dyes appears more suitable, since they exhibit higher sensitivity to small bioelectrical changes. Similar to earlier experiments using inhibitors, the modulation of bioelectrical signals via RNAi-knockdown of genes coding for ion-transport mechanisms and gap-junction subunits resulted in distinct cytoskeletal changes. Moreover, RNAi-knockdown exerted influence on crucial processes during development of the ovary and oogenesis. Therefore, by using genetic tools, further evidence amounts for bioelectrical regulation of developmental processes via control of both signalling pathways and cytoskeletal organisation.

## Methods

### Fly stocks

For FC-specific expression of the V_mem_-sensor ArcLight (Bloomington stock #51056) and the pH_i_-sensor pHluorin-Moesin (Bloomington stock #44594), respectively, the *tj*-Gal4 driver line (gift from S. Roth and O. Karst, Köln, Germany) was used. This driver line was also used for RNAi soma-knockdown experiments. For RNAi germline-knockdown experiments (controls), we used the MTD-Gal4 driver line (*w; Sco/CyO; MTD-Gal4*; gift from P. Becker, München, Germany), a mat-tub-Gal4-GeneSwitch driver line (*w; mat-tub-Gal4-GeneSwitch/CyO; +*) and a mat-tub-Gal4 driver line (*w; mat-tub-Gal4/CyO; +*; N. Lowe and D. St. Johnston, Cambridge, UK; both gifts from S. Huelsmann, Tübingen, Germany), respectively. RNAi-lines from the Vienna *Drosophila* Resource Center (VDRC [73]; stocks #v17043, #v40953, #v46553, #v47073, #v4642, #v7245 and #v8549; see Table 1 and Additional file: Table S1) were gifts from A. Voigt (Aachen, Germany). Flies carrying UAS-lhRNA- or UAS-shRNA-constructs (RNAi-lines from the Transgenic RNAi Project, TRiP [74]) were obtained from the Bloomington *Drosophila* Stock Center (in Valium10 vector: stocks #27034, #25885 and #28589; in Valium20 vector: stocks #39053, #40884, #40923, #42645, #44048, #51877, #53994 and #60112; see Table 1 and Additional file: Table S1). To verify the expression patterns of the used Gal4-drivers, UAST-GFP females (*w; UAST-gfp; +*; gift from S. Huelsmann, Tübingen, Germany) were crossed with males carrying the soma driver (see Fig. 1a), whereas females of all germline drivers were crossed with UASp-myrGFP males (Bloomington stock #58721; see Fig. 5c-e). Flies were reared at 25 °C on standard food with additional fresh yeast.

### Preparation of follicles

Female flies were killed, 2–3 days old ovaries were dissected, and single follicles of vitellogenic stages S8-S12 were isolated as described previously [5, 16, 17]. Dissection and cytoskeletal staining were carried out in *Drosophila* phosphate buffered saline [75], whereas inhibition experiments and morphological analysis were carried out in R-14 Medium [75, 76].

### Optical sectioning of living follicles

Single follicles of S8-S12, expressing either ArcLight or pHluorin-Moesin, were imaged in R-14 medium on a Zeiss AxioImager.M2 structured illumination microscope (SIM), equipped with a Zeiss ApoTome and a Zeiss AxioCamMRm camera, using a × 20/0.5 objective. Median optical sections were produced, and ImageJ (NIH, USA) was used to generate pseudocolour images as described previously [5, 16, 17].

### Inhibition experiments

All S10B-follicles of a single fly (approximately 10–20), expressing either ArcLight or pHluorin-Moesin, were divided into a control group and an experimental group. Inhibition was performed for 20 min in R-14 medium containing one of the following inhibitors of ion-transport mechanisms (cf. [5, 16]): Na^+^/H^+^-exchangers (NHE) and amiloride-sensitive Na^+^-channels were blocked with amiloride (Sigma-Aldrich, Germany; 1 mM; dissolved in dimethyl sulfoxide; DMSO), V-ATPases were blocked with concanamycin A (Biomol, Germany; 1 or 2.5 μM; dissolved in DMSO), ATP-sensitive K^+^-channels were blocked with glibenclamide (Biomol; 250 μM; dissolved in DMSO), voltage-dependent L-type Ca^2+^-channels were blocked with verapamil-HCl (Sigma-Aldrich; 1 mM; dissolved in 70% ethanol), Cl^−^-channels were blocked with 9-anthroic acid (Sigma-Aldrich; 1 mM; dissolved in DMSO), and Na^+^/K^+^/2Cl^−^-cotransporters were blocked with furosemide (Sigma-Aldrich; 1.5 mM; dissolved in DMSO), respectively. R-14 medium containing 0.25–1% v/v ethanol or DMSO was used in control experiments. Immediately after incubation, groups of three to seven follicles were imaged in covered glass block dishes on a Zeiss Axiovert 200 wide-field fluorescence microscope (WFM), equipped with a Hamamatsu Orca ER camera, using a × 10 objective as described previously [16]. During the respective experiments with either the V_mem_- or the pH_i_-sensor, exposure time and other settings remained unchanged.

### Quantification of fluorescence intensities

Original grey-scale WFM-images (Fig. 1d) were used to measure, with ImageJ, the fluorescence intensity (“mean grey value”) in the columnar FE of each follicle. The values of control follicles were averaged; then values of control and treated follicles were normalised to the mean of the control group. For each inhibitor, the experiment was repeated at least four times. To consider the variability between follicles, all normalised values of the same treatment were averaged (*relative intensity*, Fig. 2). To consider the variability between experiments, for each treatment a *mean intensity ratio* (Figs. 3 and 4) of the mean values of the experimental and the control groups (*inhibited/control*) of four repetitions was calculated. The mean values were compared using either an unpaired t-test (Fig. 2) or a one-sample t-test (Figs. 3 and 4). Microsoft Excel and GraphPad Prism were used for statistical analysis, and GraphPad Prism was used for data presentation.

### RNAi-knockdown screen

Candidate genes of ion-transport mechanisms and gap-junction subunits showing enriched ovary expression (with respect to the signal in whole flies) were selected according to the FlyAtlas 2 Gene Expression Database (http://flyatlas.gla.ac.uk/; cf. [52]). In a first experiment, the respective VDRC UAS-strains were used for RNAi in the FE. Since these RNAi-constructs (with the exception of #v46553) had no effects on either ovary morphology or oogenesis (for summary, see Additional file: Table S1), the screen was repeated using TRiP UAS-lhRNA- and UAS-shRNA-strains. Short hairpins (sh) embedded into a micro-RNA backbone are known to be very effective for knockdown in both germline and soma [74]. Males of the driver lines mat-tub-Gal4-GeneSwitch [58], mat-tub-Gal4.VP16, MTD-Gal4 or *tj*-Gal4 were crossed with UAS-lhRNA or UAS-shRNA females (in Valium10 vector [68] for soma knockdown, or in the very effective Valium20 vector [74] for soma and germline knockdown). F1 females, reared at 25 °C for 3 days on standard medium with additional fresh yeast, were dissected (*n* ≥ 10 flies for each strain). Ovaries from transcriptionally almost inactive *mat-tub-Gal4-GeneSwitch > UAS-lhRNA* flies or *mat-tub-Gal4-GeneSwitch > UAS-shRNA* flies were used as controls.

### Staining of microfilaments

Follicles of wt and *ork1*-knockdown (BL53994) flies were fixed and stained with phalloidin-FluoProbes 550A (Interchim, France) as described previously [5, 17, 19]. Thereafter, the follicles were mounted in Fluoromount G (Interchim) and viewed using SIM and a × 40/1.3 oil objective. Tangential optical sections of various stages (*n* = 27 *ork1*-follicles) were produced as described [5, 17].

### Staining of microtubules

Follicles of wt and *ork1*-knockdown (BL53994) flies were fixed, incubated with a monoclonal antibody against acetylated α-tubulin (6-11B-1; Santa Cruz Biotechnology, USA), and stained as described in detail previously [5, 17]. Thereafter, the follicles were mounted and analysed as described above using tangential optical sections (*n* = 18 *ork1*-follicles). Control follicles were treated without primary antibody.

### Nuclear staining

Ovaries of knockdown flies showing reduced size were fixed as described above and stained with 0.2 μg/ml DAPI (4′,6-diamidino-2-phenylindole; Sigma-Aldrich). Thereafter, the ovaries were mounted and viewed as described above using a × 20/0.5 or a × 40/1.3 oil objective and WFM (*n* = 7–8 ovaries per strain).

## Supplementary information

**Additional file 1: Table S1.** Summary of candidate genes showing no effects in RNAi-knockdown screen. Data corresponding to Table 1.

## Data Availability

The datasets used during the current study are available from the corresponding author on reasonable request.

## Abbreviations

a-p: Anteroposterior
bMF: Basal microfilaments
5-CFDA,AM: 5-Carboxyfluorescein diacetate, acetoxymethyl ester
cFC: Centripetal follicle cells
DAPI: 4′,6-Diamidino-2-phenylindole
DiBAC_4_(3): Bis-(1,3-dibutylbarbituric acid) trimethine oxonol
DMSO: Dimethyl sulfoxide
d-v: Dorsoventral
FC: Follicle cells
FE: Follicular epithelium
GEVI: Genetically-encoded voltage-indicator
MF: Microfilaments
mFC: Mainbody follicle cells
MT: Microtubules
NC: Nurse cells
Ooc: Oocyte
pFC: Posterior follicle cells
pH_i_: Intracellular pH
S: Stage
SIM: Structured-illumination microscopy
V_mem_: Membrane potential
vFC: Ventral FC
VSD: Voltage-sensing domain
WFM: Wide-field microscopy
wt: Wild-type

## References

[1] Chang F, Minc N (2014). Electrochemical control of cell and tissue polarity. Annu Rev Cell Dev Biol.

[2] McLaughlin KA, Levin M (2018). Bioelectric signaling in regeneration: mechanisms of ionic controls of growth and form. Dev Biol.

[3] Krüger J, Bohrmann J (2015). Bioelectric patterning during oogenesis: stage-specific distribution of membrane potentials, intracellular pH and ion-transport mechanisms in *Drosophila* ovarian follicles. BMC Dev Biol.

[4] Ulmschneider B, Grillo-Hill BK, Benitez M, Azimova DR, Barber DL, Nystul TG (2016). Increased intracellular pH is necessary for adult epithelial and embryonic stem cell differentiation. J Cell Biol.

[5] Weiß I, Bohrmann J (2019). Electrochemical gradients are involved in regulating cytoskeletal patterns during epithelial morphogenesis in the *Drosophila* ovary. BMC Dev Biol.

[6] Benitez M, Tatapudy S, Liu Y, Barber DL, Nystul TG (2019). *Drosophila* anion exchanger 2 is required for proper ovary development and oogenesis. Dev Biol.

[7] George LF, Pradhan SJ, Mitchell D, Josey M, Casey J, Belus MT, Fedder KN, Dahal GR, Bates EA (2019). Ion channel contributions to wing development in *Drosophila melanogaster*. G3.

[8] Plaster NM, Tawil R, Tristani-Firouzi M, Canún S, Bendahhou S, Tsunoda A, Donaldson MR, Iannaccone ST, Brunt E, Barohn R, Clark J, Deymeer F, George AL, Fish FA, Hahn A, Nitu A, Ozdemir C, Serdaroglu P, Subramony SH, Wolfe G, Fu YH, Ptácek LJ (2001). Mutations in Kir2.1 cause the developmental and episodic electrical phenotypes of Andersen’s syndrome. Cell.

[9] Splawski I, Timothy KW, Sharpe LM, Decher N, Kumar P, Bloise R, Napolitano C, Schwartz PJ, Joseph RM, Condouris K, Tager-Flusberg H, Priori SG, Sanguinetti MC, Keating MT (2004). Ca(V)1.2 calcium channel dysfunction causes a multisystem disorder including arrhythmia and autism. Cell.

[10] Harguindey S, Reshkin SJ, Orive G, Arranz JL, Anitua E (2007). Growth and trophic factors, pH and the Na^+^/H^+^ exchanger in Alzheimer’s disease, other neurodegenerative diseases and cancer: new therapeutic possibilities and potential dangers. Curr Alzheimer Res.

[11] Simons C, Rash LD, Crawford J, Ma L, Cristofori-Armstrong B, Miller D, Ru K, Baillie GJ, Alanay Y, Jacquinet A, Debray FG, Verloes A, Shen J, Yesil G, Guler S, Yuksel A, Cleary JG, Grimmond SM, McGaughran J, King GF, Gabbett MT, Taft RJ (2015). Mutations in the voltage-gated potassium channel gene KCNH1 cause Temple-Baraitser syndrome and epilepsy. Nat Genet.

[12] White KA, Grillo-Hill BK, Barber DL (2017). Cancer cell behaviors mediated by dysregulated pH dynamics at a glance. J Cell Sci.

[13] Simons M, Gault WJ, Gotthardt D, Rohatgi R, Klein TJ, Shao Y, Lee HJ, Wu AL, Fang Y, Satlin LM, Dow JT, Chen J, Zheng J, Boutros M, Mlodzik M (2009). Electrochemical cues regulate assembly of the Frizzled/Dishevelled complex at the plasma membrane during planar epithelial polarization. Nat Cell Biol.

[14] Levin M (2014). Endogenous bioelectrical networks store non-genetic patterning information during development and regeneration. J Physiol.

[15] Levin M (2014). Molecular bioelectricity: how endogenous voltage potentials control cell behavior and instruct pattern regulation in vivo. Mol Biol Cell.

[16] Weiß I, Bohrmann J (2019). Electrochemical patterns during *Drosophila* oogenesis: ion-transport mechanisms generate stage-specific gradients of pH and membrane potential in the follicle-cell epithelium. BMC Dev Biol.

[17] Schotthöfer SK, Bohrmann J (2020). Bioelectrical and cytoskeletal polarity are linked to altered axial polarity in the follicular epithelium of the *Drosophila* mutant *gurken*. BMC Dev Biol.

[18] Viktorinová I, König T, Schlichting K, Dahmann C (2009). The cadherin Fat2 is required for planar cell polarity in the *Drosophila* ovary. Development.

[19] Gutzeit HO (1990). The microfilament pattern in the somatic follicle cells of mid-vitellogenic ovarian follicles of *Drosophila*. Eur J Cell Biol.

[20] Bateman J, Reddy R, Saito H, van Vactor D (2001). The receptor tyrosine phosphatase Dlar and integrins organize actin filaments in the *Drosophila* follicular epithelium. Curr Biol.

[21] Frydman HM, Spradling AC (2001). The receptor-like tyrosine phosphatase lar is required for epithelial planar polarity and for axis determination within *Drosophila* ovarian follicles. Development.

[22] Gutzeit HO, Eberhardt W, Gratwohl E (1991). Laminin and basement membrane-associated microfilaments in wild-type and mutant *Drosophila* ovarian follicles. J Cell Sci.

[23] Bohrmann J, Dorn A, Sander K, Gutzeit H (1986). The extracellular electrical current pattern and its variability in vitellogenic *Drosophila* follicles. J Cell Sci.

[24] Bohrmann J, Huebner E, Sander K, Gutzeit H (1986). Intracellular electrical potential measurements in *Drosophila* follicles. J Cell Sci.

[25] Bohrmann J (1991). Potassium uptake into *Drosophila* ovarian follicles: relevance to physiological and developmental processes. J Insect Physiol.

[26] Bohrmann J, Heinrich UR (1994). Localisation of potassium pumps in *Drosophila* ovarian follicles. Zygote.

[27] Bohrmann J, Braun B (1999). Na,K-ATPase and V-ATPase in ovarian follicles of *Drosophila melanogaster*. Biol Cell.

[28] Munley SM, Kinzeler S, Lizzano R, Woodruff RI (2009). Fractional contribution of major ions to the membrane potential of *Drosophila melanogaster* oocytes. Arch Insect Biochem Physiol.

[29] Hermle T, Saltukoglu D, Grünewald J, Walz G, Simons M (2010). Regulation of Frizzled-dependent planar polarity signaling by a V-ATPase subunit. Curr Biol.

[30] Zelle KM, Lu B, Pyfrom SC, Ben-Shahar Y (2013). The genetic architecture of degenerin/epithelial sodium channels in *Drosophila*. G3.

[31] Mano I, Driscoll M (1999). DEG/ENaC channels: a touchy superfamily that watches its salt. BioEssays.

[32] Adams CM, Anderson MG, Motto DG, Price MP, Johnson WA, Welsh MJ (1998). Ripped pocket and pickpocket, novel *Drosophila* DEG/ENaC subunits expressed in early development and in mechanosensory neurons. J Cell Biol.

[33] Kellenberger S, Schild L (2002). Epithelial sodium channel/degenerin family of ion channels: a variety of functions for a shared structure. Physiol Rev.

[34] Darboux I, Lingueglia E, Champigny G, Coscoy S, Barbry P, Lazdunski M (1998). dGNaC1, a gonad-specific amiloride-sensitive Na^+^-channel. J Biol Chem.

[35] Wieczorek H, Putzenlechner M, Zeiske W, Klein U (1991). A vacuolar-type proton pump energizes K^+^/H^+^-antiport in an animal plasma membrane. J Biol Chem.

[36] Allan AK, Du J, Davies SA, Dow JAT (2005). Genome-wide survey of V-ATPase genes in *Drosophila* reveals a conserved renal phenotype for lethal alleles. Physiol Genomics.

[37] Lautemann J, Bohrmann J (2016). Relating proton pumps with gap junctions: colocalization of ductin, the channel-forming subunit c of V-ATPase, with subunit a and with innexins 2 and 3 during *Drosophila* oogenesis. BMC Dev Biol.

[38] Vaccari T, Duchi S, Cortese K, Tacchetti C, Bilder D (2010). The vacuolar ATPase is required for physiological as well as pathological activation of the Notch receptor. Development.

[39] Tognon E, Kobia F, Busi I, Fumagalli A, de Masi F, Vaccari T (2016). Control of lysosomal biogenesis and Notch-dependent tissue patterning by components of the TFEB-VATPase axis in *Drosophila melanogaster*. Autophagy.

[40] Bohrmann J, Haas-Assenbaum A (1993). Gap junctions in ovarian follicles of *Drosophila melanogaster*: inhibition and promotion of dye-coupling between oocyte and follicle cells. Cell Tissue Res.

[41] Bauer R, Löer B, Ostrowski K, Martini J, Weimbs A, Lechner H, Hoch M (2005). Intercellular communication: the *Drosophila* innexin multiprotein family of gap junction proteins. Chem Biol.

[42] Phelan P (2005). Innexins: members of an evolutionarily conserved family of gap-junction proteins. Biochim Biophys Acta.

[43] Stebbings LA, Todman MG, Phillips R, Greer CE, Tam J, Phelan P, Jacobs K, Bacon JP, Davies JA (2002). Gap junctions in *Drosophila*: developmental expression of the entire innexin gene family. Mech Dev.

[44] Bohrmann J, Zimmermann J (2008). Gap junctions in the ovary of *Drosophila melanogaster*: localization of innexins 1, 2, 3 and 4 and evidence for intercellular communication via innexin-2 containing channels. BMC Dev Biol.

[45] Lin MZ, Schnitzer MJ (2016). Genetically encoded indicators of neuronal activity. Nat Neurosci.

[46] Kulkarni RU, Miller EW (2017). Voltage imaging: pitfalls and potential. Biochemistry..

[47] Fishilevich E, Fitzpatrick JAJ, Minden JS (2010). pHMA, a pH-sensitive GFP reporter for cell engulfment, in *Drosophila* embryos, tissues, and cells. Dev Dyn.

[48] Han J, Burgess K (2010). Fluorescent indicators for intracellular pH. Chem Rev.

[49] Adams DS, Levin M (2012). Measuring resting membrane potential using the fluorescent voltage reporters DiBAC_4_(3) and CC2-DMPE. Cold Spring Harb Protoc.

[50] Horne-Badovinac S, Hill J, Gerlach G, Menegas W, Bilder D (2012). A screen for round egg mutants in *Drosophila* identifies tricornered, furry, and misshapen as regulators of egg chamber elongation. G3.

[51] Giannakou ME, Dow JA (2001). Characterization of the *Drosophila melanogaster* alkali-metal/proton exchanger (NHE) gene family. J Exp Biol.

[52] Robinson SW, Herzyk P, Dow JAT, Leader DP (2013). FlyAtlas: database of gene expression in the tissues of *Drosophila melanogaster*. Nucleic Acids Res.

[53] Hoffmann EK (1982). Anion exchange and anion-cation co-transport systems in mammalian cells. Philos Trans R Soc Lond Ser B Biol Sci.

[54] Sherwood AC, John-Alder K, Sanders MM (1988). Characterization of chloride uptake in *Drosophila* Kc cells. J Cell Physiol.

[55] Huss M, Ingenhorst G, König S, Gassel M, Dröse S, Zeeck A, Altendorf K, Wieczorek H (2002). Concanamycin A, the specific inhibitor of V-ATPases, binds to the V(o) subunit c. J Biol Chem.

[56] Moreno SN, Zhong L, Lu HG, Souza WD, Benchimol M (1998). Vacuolar-type H^+^-ATPase regulates cytoplasmic pH in *Toxoplasma gondii* tachyzoites. Biochem J.

[57] Ball AJ, Flatt PR, McClenaghan NH (2000). Desensitization of sulphonylurea- and nutrient-induced insulin secretion following prolonged treatment with glibenclamide. Eur J Pharmacol.

[58] Osterwalder T, Yoon KS, White BH, Keshishian H (2001). A conditional tissue-specific transgene expression system using inducible GAL4. Proc Natl Acad Sci U S A.

[59] Cao G, Platisa J, Pieribone VA, Raccuglia D, Kunst M, Nitabach MN (2013). Genetically targeted optical electrophysiology in intact neural circuits. Cell.

[60] Chintapalli VR, Wang J, Herzyk P, Davies SA, Dow JAT (2013). Data-mining the FlyAtlas online resource to identify core functional motifs across transporting epithelia. BMC Genomics.

[61] Davies SA, Goodwin SF, Kelly DC, Wang Z, Sozen MA, Kaiser K, Dow JAT (1996). Analysis and inactivation of *vha55*, the gene encoding the vacuolar ATPase B subunit in *Drosophila melanogaster* reveals a larval lethal phenotype. J Biol Chem.

[62] Du J, Kean L, Allan AK, Southall TD, Davies SA, McInerny CJ, Dow JAT (2006). The SzA mutations of the B subunit of the *Drosophila* vacuolar H^+^-ATPase identify conserved residues essential for function in fly and yeast. J Cell Sci.

[63] Eaton S, Martin-Belmonte F (2014). Cargo sorting in the endocytic pathway: a key regulator of cell polarity and tissue dynamics. Cold Spring Harb Perspect Biol.

[64] Tazuke SI, Schulz C, Gilboa L, Fogarty M, Mahowald AP, Guichet A, Ephrussi A, Wood CG, Lehmann R, Fuller MT (2002). A germline-specific gap junction protein required for survival of differentiating early germ cells. Development.

[65] Akiyama T (2002). Mutations of *stonewall* disrupt the maintenance of female germline stem cells in *Drosophila melanogaster*. Develop Growth Differ.

[66] Sahu A, Ghosh R, Deshpande G, Prasad M (2017). A gap junction protein, Inx2, modulates calcium flux to specify border cell fate during *Drosophila* oogenesis. PLoS Genet.

[67] Bohrmann J (1993). Antisera against a channel-forming 16 kDa protein inhibit dye-coupling and bind to cell membranes in *Drosophila* ovarian follicles. J Cell Sci.

[68] Smendziuk CM, Messenberg A, Vogl AW, Tanentzapf G (2015). Bi-directional gap junction-mediated soma-germline communication is essential for spermatogenesis. Development.

[69] Duhart JC, Parsons TT, Raftery LA (2017). The repertoire of epithelial morphogenesis on display: progressive elaboration of *Drosophila* egg structure. Mech Dev.

[70] Tucker JB, Meats M (1976). Microtubules and control of insect egg shape. J Cell Biol.

[71] Lalevée N, Monier B, Sénatore S, Perrin L, Sémériva M (2006). Control of cardiac rhythm by ORK1, a *Drosophila* two-pore domain potassium channel. Curr Biol.

[72] Zhang X, Zheng Y, Ren Q, Zhou H (2017). The involvement of potassium channel ORK1 in short-term memory and sleep in *Drosophila*. Medicine.

[73] Dietzl G, Chen D, Schnorrer F, Su KC, Barinova Y, Fellner M, Gasser B, Kinsey K, Oppel S, Scheiblauer S, Couto A, Marra V, Keleman K, Dickson BJ (2007). A genome-wide transgenic RNAi library for conditional gene inactivation in *Drosophila*. Nature.

[74] Ni JQ, Zhou R, Czech B, Liu LP, Holderbaum L, Yang-Zhou D, Shim HS, Tao R, Handler D, Karpowicz P, Binari R, Booker M, Brennecke J, Perkins LA, Hannon GJ, Perrimon N (2011). A genome-scale shRNA resource for transgenic RNAi in *Drosophila*. Nat Methods.

[75] Robb JA (1969). Maintenance of imaginal discs of *Drosophila melanogaster* in chemically defined media. J Cell Biol.

[76] Bohrmann J (1991). In vitro culture of *Drosophila* ovarian follicles: the influence of different media on development, RNA synthesis, protein synthesis and potassium uptake. Roux’s Arch Dev Biol.

