# Supplementary material for: Analysing bioelectrical phenomena in the Drosophila ovary with genetic tools: tissue-specific expression of sensors for membrane potential and intracellular pH, and RNAi-knockdown of mechanisms involved in ion exchange
Source: BMC Dev Biol. 2020 Jul 8;20:15. doi: 10.1186/s12861-020-00220-6 (PMC7341674; doi:10.1186/s12861-020-00220-6)
Supplement: Supplementary file 1 — Additional file 1: Table S1. Summary of candidate genes showing no effects in RNAi-knockdown screen. Data corresponding to Table 1. [file 12861_2020_220_MOESM1_ESM.pdf]

Table S1: Summary of candidate genes showing no effects in RNAi-knockdown screen. Data corresponding to Table 1.

Stock ID	Gene name	Protein function	Phenotype Soma	Germline
**Proton pumps**				
BL40923	*vha16-1* (ductin)	V-type H^+-ATPase subunit	no effects	no effects*◊
**Chloride channels**				
BL27034	*clc-c*	Chloride channel	no effects	not determined
VDRC4642	*clc-b*	Chloride channel	no effects	not determined
**NHE**				
BL28589	*nhe1*	Sodium-proton exchanger	no effects	not determined
VDRC7245	*nhe1*	Sodium-proton exchanger	no effects	not determined
**Sodium channels**				
VDRC8549	*rpk (dGNaC1)*	DEG/epithelial sodium channel	no effects	not determined
**Potassium channels**				
BL25885	*ork1*	Open-rectifier potassium channel	no effects	not determined
VDRC40953	*ork1*	Open-rectifier potassium channel	no effects	not determined
VDRC17043	*mri*	Voltage-gated potassium channel	no effects	not determined
**Calcium channels**				
VDRC47073	*stim*	Calcium-induced calcium release activity/ store-operated calcium channel	no effects	not determined
**Innexins**				
BL42645	*inx2*	Gap-junction subunit	no effects	no effects*◊

At least 10 females were scored for each strain.
BL, Bloomington *Drosophila* Stock Center number.
VDRC, Vienna *Drosophila* Resource Center number.
Soma driver: *tj*-Gal4, * germline driver: MTD-Gal4, ◊ germline driver: mat-tub-Gal4.
